# Neurofibromatosis Type-2 presenting with vision impairment

**DOI:** 10.12669/pjms.39.2.6813

**Published:** 2023

**Authors:** Sultan Abdulwadoud Alshoabi

**Affiliations:** Sultan Abdulwadoud Alshoabi, Department of Diagnostic Radiology Technology, College of Applied Medical Sciences, Taibah University, Almadinah Almunawwarah, Kingdom of Saudi Arabia

**Keywords:** Neurofibromatosis Type-2, Visual impairment, Vestibular schwannoma, Multiple meningiomas, Tinnitus

## Abstract

Neurofibromatosis Type-2 (NF2) is an autosomal dominant genetic tumour-predisposing condition caused by mutations in the *NF2* gene located on chromosome 22q12. It is characterized by multiple benign tumours of the central and peripheral nervous systems and meninges, causing high morbidity. Herein, presentation of a rare case of NF2 in a 36-year-old female who presented with right eye visual disturbances, followed by tinnitus with hearing impairment. The visual disturbance developed into blindness. Magnetic resonance imaging (MRI) was performed, which showed a right-side cerebellopontine angle vestibular schwannoma and multiple meningiomas around the brain. According to the MRI findings, the patient was diagnosed with NF2. This case report aims to elucidate the importance of early brain imaging in any visual disturbances in young adults and to highlight the key role of medical imaging in the diagnosis of rare cases. Moreover, this describe the MRI features and the diagnostic accuracy for the tumours occurring in NF2 in detail.

## INTRODUCTION

Neurofibromatoses are a group of genetic disorders that cause multiple tumours in major body systems, particularly the nervous system. Neurofibromatosis type 1 (NF1) is the most common condition caused by *NF1* *gene* mutations and has the most variable phenotype with a high risk of malignant tumours.^1^,^2^ Neurofibromatosis Type-2 (NF2) is an autosomal dominant genetic condition caused by mutations in the *NF2* *gene* located on chromosome 22q12 with genotype-phenotype relationships.^3^ NF2 is a rare condition with an incidence of 1: 33,000-40,000 live births annually. It is characterized by the development of multiple benign tumours of the central and peripheral nervous system and meninges, presenting with variable and unspecific clinical features and causing high morbidity.^4^

In the literature, many published cases of NF2 with typical presentations were found. Conversely, cases of NF2 with unilateral visual and unilateral hearing impairments are uncommon This report details a rare case of NF2 that presented with unilateral right-side visual disturbances and developed into blindness and hearing impairment. This case report aims to elucidate the importance of early brain imaging in any visual disturbances in young adults and highlight the key role of medical imaging in diagnosing rare cases. Moreover, description of the magnetic resonance imaging (MRI) features of NF2 tumours was done in detail.

## CASE PRESENTATION

A 36-year-old female presented with recurrenting transient attacks of amblyopia in the right eye. The patient saw an ophthalmologist who performed fundoscopy and found a right optic nerve oedema with blood vessels tortuosity and a normal left-side fundus. After completing computed tomography of the head, the diagnosis was optic neuritis. The patient underwent medical treatment and the condition improved for one month before the patient relapsed with a frontal headache. After nine months, the patient began to complain of continuous tinnitus in the right ear, with hearing impairment.

**Fig.1:**
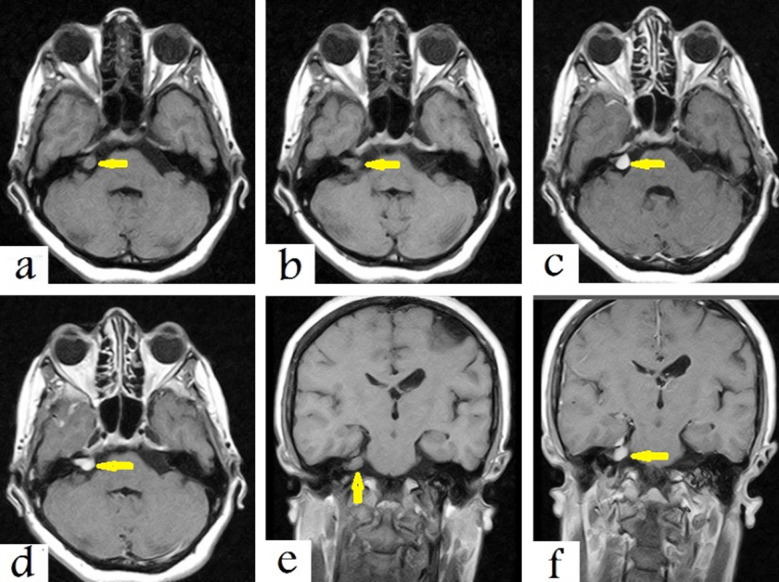
Selected images of a brain MRI, a, b) Axial T1-weighted images show a well-defined Isointense mass in the right cerebellopontine angle (arrows) with meatal extension along the right vestibular nerve, c, d) Contrast enhanced T1-weighted images show avid mass enhancement after contrast administration (arrows), e, f) Coronal T1-weighted images pre- and post-contrast show avid mass enhancement after contrast, which is typical feature of right vestibular schwannoma (arrows)..

After four months, an MRI was performed which showed a well-defined mass in the right cerebellopontine angle with extension along the right vestibular nerve. The mass appeared to be of isointense signal intensity on T1-weighed images (TWIs) with avid enhancement after contrast administration on all axial (Fig.1a, b, c, and d) and coronal (Fig.1e and f) sections of the MRI. These are typical features of vestibular schwannoma.

**Fig.2:**
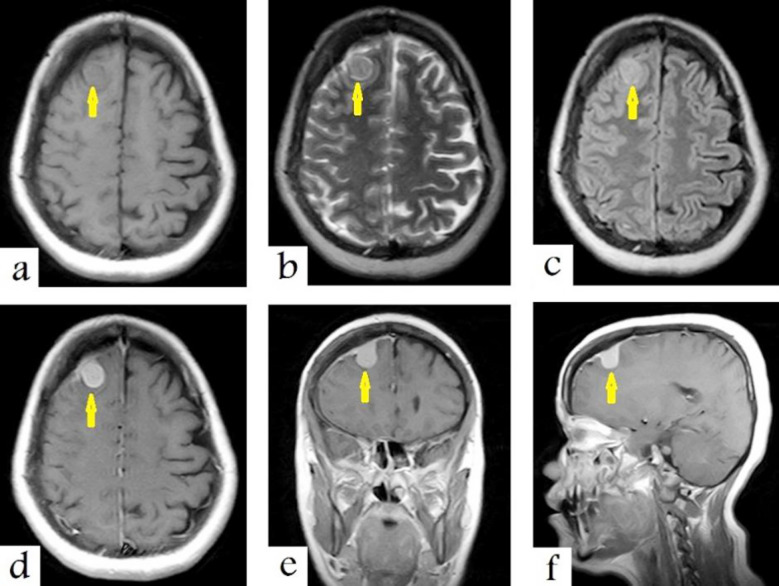
Selected images of the brain MRI show a well-defined extra-axial tumuor of approximately 26×17 mm in the right frontal lobe. It appears isointense on T1-weighted images (a), sight hyperintense on T2-weighted images (b) and fluid-attenuation inversion recovery (FLAIR), (c) with avid enhancement and a dural-base and tail on T1-weighted images after contrast administration, (d, e and f). This strongly suggests right-side convexity meningioma (arrows).

The brain MRI shows a well-defined extra-axial tumuor of approximately 26×17 mm in the right frontal lobe, which appears isointense on T1WIs, (Fig.2a), sight hyperintense on T2WIs (Fig.2b) and fluid-attenuation inversion recovery (FLAIR) (Fig.2c) with avid enhancement after contrast administration. It has a broad dural-base and tail (Fig.2d, Fig.2e and f). The above findings strongly suggest right-side convexity meningioma.

**Fig.3:**
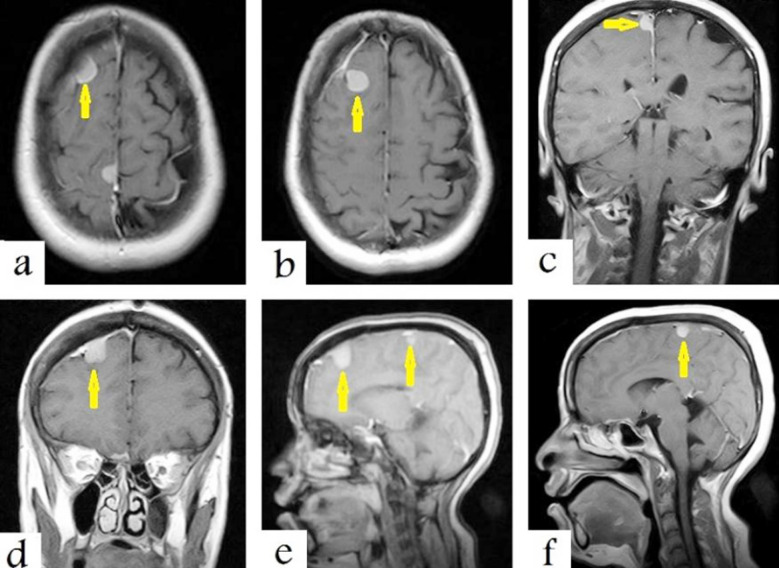
Selected T1-weighted MRI images with contrast administration show other brain tumours with avid enhancement on axial (a and b), coronal (c and d), and sagittal (e and f) sections, which are typical in images of convexity and parasagittal meningiomas (arrows).

The brain MRI T1WIs with contrast administration showed other brain tumours with avid enhancement and dural-bases and tails after contrast administration on all axial (Fig.3a and b), coronal (Fig.3c and d), and sagittal (Fig.3e and f) images, which are typical in images of convexity and parasagittal meningiomas.

In the five-month follow up, the patient complained of complete vision loss in the right eye with tinnitus and hearing impairment in the right ear with headaches, especially on the right side of the head. MRI showed the same findings, with no appreciable changes.

## DISCUSSION

NF2’s clinical presentation is variable and non-specific, which make early diagnosis challenge. Our patient presented with right eye visual impairment followed by hearing impairment. These symptoms align with a study by Forde et al. that reported vision loss as the most common presenting symptom (23%), followed by hearing loss (20.9%).^5^ Another study reported that NF2’s most frequent presenting clinical manifestations in children were ophthalmological abnormalities (49%), followed by cutaneous manifestations (40%), vestibular schwannoma-related symptoms (21%), and other neurological deficits (33%).^4^

Our patient presented with gradual visual impairment in her right eye and medical imaging found no visible nerve tumours. Gugel et al. reported that visual impairment/loss is the most common ophthalmological symptom in adult NF2 patients. The cause of visual impairment is idiopathic, optic nerve sheath meningioma-associated, cataracts, or retinal hamartomas/maculopathy.^6^ After nine months of visual impairment, tinnitus with hearing impairment occurred in our patient. This is compatible with Lan et al., who reported that most NF2 adult patients initially presented with unilateral hearing loss with accompanying or preceding tinnitus.^7^

The pathophysiology of NF2 hearing loss is not fully understood and may be attributable to several complex factors including; direct mechanical compression of the adjacent neurovascular structures, tumour secreting factors that cause a loss of hair cells, and elevated perilymphatic protein level that induce cochlear dysfunction.^6^ A study by Asthagiri et al. indicated that elevated intralabyrinthine perilymphatic proteins may underlie hearing loss, and labyrinthine high signal intensity on MRI in normal hearing ears may serve as an imaging biomarker for developing hearing loss.^8^

NF2 is a tumuor predisposition syndrome characterized by the growth of multiple tumours in the nervous system, which commonly present in young adults as multiple cranial neuropathies. Schwannomas and meningiomas are a major concern in NF2; however, ependymomas are rare [9]. Our patient is a young adult female who presented with optic (cranial nerve-2) and vestibular (part of cranial nerve-8) neuropathies with right-side vestibular schwannoma and multiple brain meningiomas.

Regarding the pathophysiology of multiple tumor growth, merlin is a tumuor suppressor protein that acts as a scaffolding protein to regulate multiple signaling pathways and integrates extracellular signals to modulate cell morphology, motility, proliferation and survival. Merlin is encoded by the *NF2 gene*, and loss of function mutations or deletions of the *NF2 gene* cause NF2 with the development of schwannomas, meningiomas and ependymomas.^10^

According to Carlson et al., high-resolution MRI with gadolinium accurately diagnoses vestibular schwannomas, even tiny (smaller than 2mm) ones. Classically presenting, cerebellopontine vestibular schwannoma is centered on the internal acoustic canal and usually show meatal extension. They appear hypo- to isointense on T1WIs, iso- to hyper intense on T2WIs, and show avid enhancement after contrast administration.^11^ In our patient, the mass in the right cerebellopontine angle meets typical imaging features of vestibular schwannoma. We depended on MRI for the diagnosis of vestibular schwannoma which is a non-invasive, safe and convenient imaging modality with 96%, 88.2%, 92.31%, 93.75% and 92.86% overall sensitivity, specificity, positive predictive value (PPV), negative predictive value (NPV), and diagnostic accuracy respectively.^12^

According to Wattas et al. Meningioma’s are slow-growing tumors arising from the meningothelial cells of the arachnoid along any of the external surfaces of the brain or the stromal arachnoid cells of the choroid plexus within the ventricular system. The most common meningioma locations include the parasagittal aspect of the cerebral hemisphere, the lateral cerebral convexity and the sphenoid wing. They typically appear as well-circumscribed extra-axial masses with iso- to slightly hypo intense relative to the grey matter on T1WIs, and iso- to slightly hyper intense on T2WIs with avid homogeneous enhancement after contrast administration.^13^ In our patient, the brain MRI shows right convexity and parasagittal meningioma’s. MRI has 82.6%, 97%, 93%, 91.6%, and 92% sensitivity, specificity, PPV, NPV, and overall diagnostic accuracy, respectively for Grade-I meningiomas.^14^

According to the current and revised Manchester criteria, (2017), NF2 can be diagnosed by: (1)-bilateral vestibular schwannoma, (2)-first-degree relative family history with NF2 and unilateral vestibular schwannoma, (3)- first-degree relative family history with NF2 or unilateral vestibular schwannoma and two of the following: meningioma, cataract, ependymoma, schwannoma, and cerebral calcification, (4)-multiple meningioma’s (≥2) and two of the following: unilateral vestibular schwannoma, cataract, ependymoma, schwannoma, and cerebral calcification, or (5)-constitutional pathogenic *NF2 gene* variant either in blood or identical in two tumours.^15^ In our patient, we found unilateral right vestibular schwannoma, multiple convexity and parasagittal meningioma’s which meet the criterion number (3) for diagnosing NF2.

NF2 is life-limiting with many morbidities. Early diagnosis of NF2 is necessary to pursue early awareness of vestibular schwannomas and optic nerve meningioma’s and to monitor for hearing or visual loss to prevent deafness or blindness. Treatment involves regular monitoring to check for any health problems-these should be treated by specialized health professionals, if necessary. After our patient diagnosis, she was referred to a neurosurgical consultation to plan her management. There have been no available follow up to update the information.

The strength of this case report is that it presents a rare medically significant syndrome with a very rare presentation, elucidate the importance of early brain imaging in any visual disturbances in young adults, and highlights the role of medical imaging in diagnosing rare cases. However, it is limited in that *NF2 gene* mutation screenings were not performed because they are unavailable in in Yemen.

## CONCLUSION

The majority of NF2 patients present with hearing impairment, which may be accompanied or preceded by tinnitus. However, vision impairment may be the first presenting symptoms, which may cause the patient to consult an ophthalmologist, delaying diagnosis.

### Learning points from this case:

1. Early medical imaging of the brain is important in any visual disturbances in children, adolescents or young adults.

2. Medical imaging of the brain is the cornerstone for diagnosing patients with NF2.

### Ethical Approval:

The patient has given informed consent, allowing the author to publish this case and these images.
